# Case Report: Lupoid cutaneous leishmaniasis mimicking verruca plana

**DOI:** 10.12688/f1000research.11591.1

**Published:** 2017-06-20

**Authors:** Emin Ozlu, Aysegul Baykan, Ozan Yaman, Ragıp Ertas, Mustafa Atasoy, Kemal Ozyurt, Abdullah Turasan, Nazan Taslıdere

**Affiliations:** 1 Department of Dermatology, School of Medicine, Duzce University, Duzce, Turkey; 2 Department of Dermatology, Kayseri Tekden Hospital, Kayseri, Turkey; 3 Department of Microbiology, Kayseri Training and Research Hospital, Kayseri, Turkey; 4 Department of Dermatology, Kayseri Training and Research Hospital, Kayseri, Turkey

**Keywords:** diagnosis, leishmaniasis, viral disease

## Abstract

Cutaneous leishmaniasis (CL) is an infectious disease caused by various species of leishmania protozoan parasites. Lupoid CL is a rare form of CL that has a stunning similarity to other granulomatous cutaneous conditions of infectious or inflammatory origin. Verruca plana, also known as a “flat wart”, is a benign proliferation of the skin resulting from infection with human papilloma virus (HPV). Herein, we presented a case of lupoid CL mimicking verruca plana on the face.

## Introduction

Leishmaniasis encompasses a group of diseases caused by the protozoan parasites of the Leishmania genus ^1^. Classical lesions of cutaneous leishmaniasis (CL) advance in the forms of papules, nodules and ulcerated lesions, and they heal with an atrophic scar over months and years ^2^. Nevertheless, CL has been seen in atypical form, including erysipeloid, lupoid, sporotrichoid, hyperkeratotic, eczematous, verrucous and impetiginized form ^3^.

Lupoid CL is one of the more rarely seen forms of CL ^4^. The incidence of lupoid CL has been reported to be 0.5 to 6.2% ^5^. This clinical presentation with a chronic course develops after acute CL infection. In this clinical form, papulonodular lesions of granulomatous and lupoid character are seen 1–2 years after the acute lesion is healed ^4^. Although there is an immune response against parasites in lupoid CL, the immune system is unable to remove the parasites altogether and thus the chronic granulomatous response continues for a long time ^6^. Many clinical presentations of lupoid CL have been reported; however, no lupoid CL mimicking verruca plana has, to our knowledge, previously been reported in the literature.

## Case report

A 9-year-old male patient presented at our clinic with multiple papular lesions located in the left cheek. He had had these lesion for three months, and they had gradually enlarged. The medical history revealed that the patient had a follow-up after diagnosis of CL located in the nose two years ago, and presented with complete regression after he was started on intralesional meglumine antimonate.

**Figure 1.**
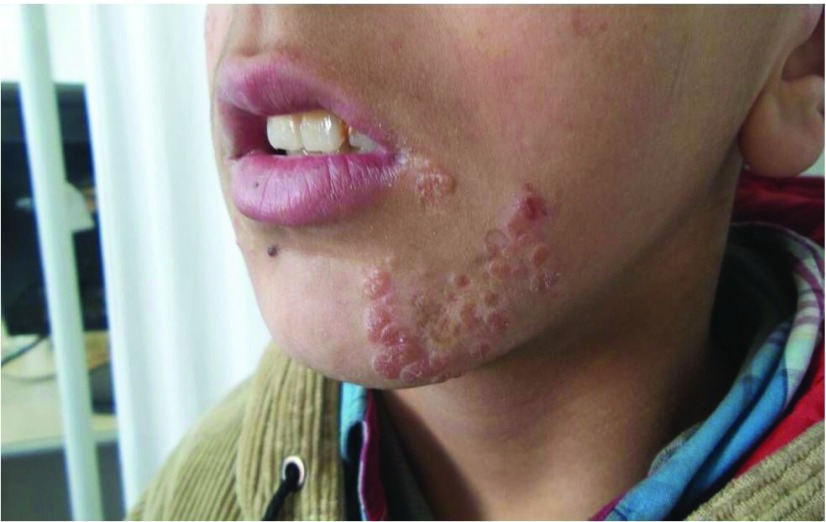
Multiple yellowish-brown coalescing papular lesions located in the patient’s left cheek.

**Figure 2.**
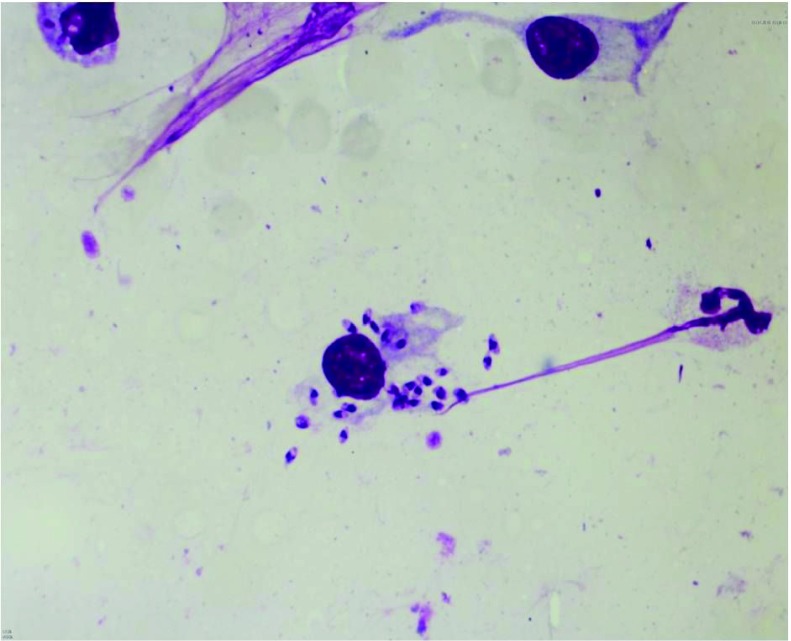
Leishmania spp. amastigotes (Giemsa staining x1000).

Dermatological examination revealed multiple, coalescing, rough, slightly elevated, yellowish-brown papular lesions 3–6 mm in diameter located in the left cheek ( Figure 1). In addition, he had large atrophic scar on the nose. Nothing of interest was noted in his family history or his laboratory tests. After staining with Giemsa, a parasitological smear showed numerous leishmania parasites in their amastigote form ( Figure 2).

**Figure 3.**
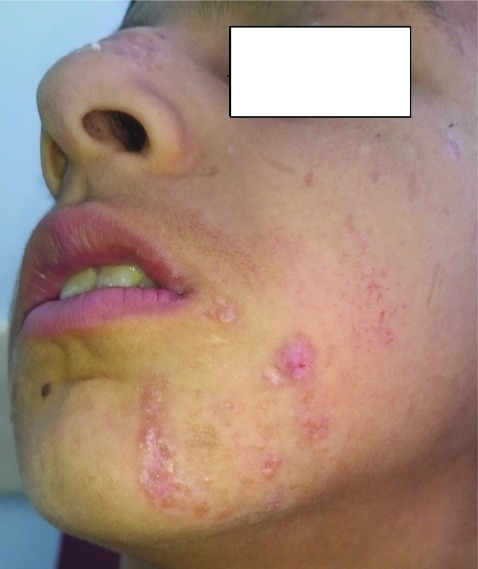
Yellowish-brown, slightly elevated papular lesions located in the left cheek after treatment.

The patient was diagnosed with lupoid CL based on his medical history, and his clinical and microscopy findings. He was started on intralesional meglumine antimonate injection per week. After the 4 sessions of treatment, significant improvement was observed in the patient’s lesions ( Figure 3).

## Discussion

CL is a parasitic disease caused by leishmania protozoa, transmitted to humans during blood sucking by infected phlebotomine sandflies ^3^. Clinical signs of CL vary from a self-limited asymptomatic presentation to life-threatening diffuse destructive lesions, depending on the type of the leishmania and the immunological state of the host. Initial lesions are frequently erythematous papules or nodular lesions ^7^.

Lupoid CL is a rare, chronic form of CL that develops following acute CL. In this clinical form, papulonodular lesions are developed at the edges of the scar months and even years after the acute lesion is healed. Papular lesions in brownish red or brownish yellow with a tendency to merge with each other, and nodules in apple-jelly consistency compose the characteristic lupoid image in lupoid CL. The lesions are sometimes squamous, crusted, and psoriasiform and may mimic lupus vulgaris. The clinical course of lupoid CL is considered to be associated with changes in the cell-mediated immunity. A possible underlying pathogenetic mechanism involves changes in Th1 and Th2 cell responses and interleukin 4 (IL-4) production ^8^. The altered host immune response then contributes to the high sensitivity to parasitic infections and extraordinary clinical presentations ^8^.

Lupoid CL has been defined as having atypical clinical properties and a chronic recurrent course. Clinically, lupoid CL may particularly resemble lupus erythematosis and lupus vulgaris ^8^. It may also resemble other granulomatous diseases of infectious or inflammatory origin and may mimic them; however, microscopic and histopathological findings are important in differentiating them from other dermatoses ^9^.

Ul Bari *et al*. ^8^ evaluated 16 patients with lupoid CL and reported 4 different morphological patterns, including psoriasiform lesions, ulcerated/crusted lesions and discoid lupus erythematosis. Douba *et al*. ^9^ analyzed 1880 patients with chronic CL. In that study, 1.4 % of 1880 patients were reported to have lesions with verrucous character ^9^. In this case report, the patient had a progressively increasing amount of groups of papular lesions that were yellowish brown in colour, in the left cheek and chin region. Clinically, the lesions suggested verruca plana; however, the microscopic evaluation of the sample obtained from the lesion revealed amastigotes and a diagnosis of lupoid CL was made. To the best of our knowledge, there is no case of lupoid CL mimicking verruca plana in the literature. In this case, it is striking that no lesion was seen around the CL scar.

Amastigotes are seen rarely in the parasitological smear in lupoid CL ^10^. In our present case, amastigotes may have been observed due to the lesions having appeared in the last three months.

There is no current protocol for efficient and accurate treatment of lupoid CL. First-line treatment involves administration of pentavalent antimony compounds ^10^. In the present case, following treatment with intralesional meglumine antimonate for 4 sessions, a significant regression in the lesions of the patient was observed; and treatment was discontinued.

## Conclusion

Lupoid CL is a rare and chronic form of CL. Lupoid CL manifest with atypical clinical and histopathological properties. It should be considered that lupoid CL may be seen as lesions similar to verruca plana.

## Consent

We obtained written informed consent from patient’s parents for the publication of the manuscript.
