# Supplementary material for: Case Report: Lupoid cutaneous leishmaniasis mimicking verruca plana
Source: F1000Res. 2017 Jun 20;6:947. [Version 1] doi: 10.12688/f1000research.11591.1 (PMC5490479; doi:10.12688/f1000research.11591.1)
Supplement: Peer review history

# Peer review: Referee response for version 1

**DOI:** 10.5256/f1000research.12521.r24237

**Published:** 2017-07-24

**Authors:** Roderick J Hay

**Affiliations:** 1 International Foundation for Dermatology, London, W1P 5HQ, UK

This is an interesting case report of an unusual variant of cutaneous leishmaniasis. It is infrequently reported and therefore worth recording. I'm not sure that these lesions resemble plane warts - they are certainly flattish but the lateral borders appear slightly curved. I would call them large flat topped papules instead.

I have read this submission. I believe that I have an appropriate level of expertise to confirm that it is of an acceptable scientific standard.

# Peer review: Referee response for version 1

**DOI:** 10.5256/f1000research.12521.r23660

**Published:** 2017-07-03

**Authors:** Mehmet Akif Dundar

**Affiliations:** 1 Department of Pediatric, Kayseri Training and Research Hospital, Kayseri, Turkey

I read the case report. The differential diagnosis of lupoid cutaneous leishmaniasis is difficult and may depend on the detection of a few Leishmania amastigotes in the histologic sections. The case provided will most likely be highly favorable for management  of this disease in Turkey. I think this case report is appropriate for publication and will contribute to the literature.

I have read this submission. I believe that I have an appropriate level of expertise to confirm that it is of an acceptable scientific standard.

# Peer review: Referee response for version 1

**DOI:** 10.5256/f1000research.12521.r23760

**Published:** 2017-06-23

**Authors:** Altay Atalay

**Affiliations:** 1 Department of Medical Microbiology, Faculty of Medicine, Erciyes University, Kayseri, Turkey

The paper titled “Case Report: Lupoid cutaneous leishmaniasis mimicking verruca plana” makes a significant contribution to the field because of its rarity. The topic is important. According to my opinion the background of the case’s history and progression described in sufficient detail and details provided of any physical examination and diagnostic tests, treatment given and outcomes are enough. In addition the case is presented  with sufficient detail to be useful for other practitioners. Figures are useful.

 In conclusion, the paper is novel and the work delivers what it promises. My overall evaluation of the paper is positive.

I have read this submission. I believe that I have an appropriate level of expertise to confirm that it is of an acceptable scientific standard.

# Peer review: Referee response for version 1

**DOI:** 10.5256/f1000research.12521.r23759

**Published:** 2017-06-23

**Authors:** Gulhan Gurel

**Affiliations:** 1 Department of Dermatology, School of Medicine, Bozok University, Yozgat, Turkey

I evaluated the case report entitled  ‘Lupoid cutaneous leishmaniasis mimicking verruca plana’. 

 Leishmania is currently endemic in 102 countries, areas or territories worldwide and 2 million new cases are recorded annually ^1^. This sentence can be added at the introduction of the article.

   

 Cutaneous leishmaniasis is still considered an important health issue in many parts of the world. Lupoid CL is a very rare form of CL. The lupoid CL mimicking the verruca plana may lead to delay in the diagnosis. It is thought that the very rare case will contribute to the literature. This case report is appropriate for publication.

I have read this submission. I believe that I have an appropriate level of expertise to confirm that it is of an acceptable scientific standard.

## References

[1] ÖzbilginAHarmanMKarakuşMBartATözSKurtÖÇavuşİPolatEGündüzCVan GoolTÖzbelY: Leishmaniasis in Turkey: Visceral and cutaneous leishmaniasis caused by Leishmania donovani in Turkey. *Acta Trop*.2017;173: 10.1016/j.actatropica.2017.05.032 90-96 10.1016/j.actatropica.2017.05.032 28587839
